# Is sham cTBS real cTBS? The effect on EEG dynamics

**DOI:** 10.3389/fnhum.2014.01043

**Published:** 2015-01-08

**Authors:** Alexander Opitz, Wynn Legon, Jerel Mueller, Aaron Barbour, Walter Paulus, William J. Tyler

**Affiliations:** ^1^Department of Clinical Neurophysiology, Georg-August-UniversityGöttingen, Germany; ^2^Center for Biomedical Imaging and Neuromodulation, Nathan Kline Institute for Psychiatric ResearchOrangeburg, NY, USA; ^3^Center for the Developing Brain, Child Mind InstituteNew York, NY, USA; ^4^Department of Physical Medicine and Rehabilitation, University of MinnesotaMinneapolis, MN, USA; ^5^School of Biomedical Engineering and Sciences, Virginia TechBlacksburg, VA, USA; ^6^Virginia Tech Carilion Research InstituteRoanoke, VA, USA; ^7^School of Biological and Health Systems Engineering, Arizona State UniversityTempe, AZ, USA

**Keywords:** TMS, EEG, electric field, sham TMS, DLPFC, evoked potentials, somatosensory

## Abstract

Increasing sensitivity of modern evaluation tools allows for the study of weaker electric stimulation effects on neural populations. In the current study we examined the effects of sham continuous theta burst (cTBS) transcranial magnetic stimulation to the left dorsolateral prefrontal cortex (DLPFC) upon somatosensory evoked potentials (SEP) and frontal-parietal phase coupling of alpha and beta bands. Sham TMS results in an induced electric field amplitude roughly 5% that of real TMS with a similar spatial extent in cortex. Both real and sham cTBS reduced the amplitude of the frontal P14-N30 SEP and increased local phase coupling in the alpha-beta frequency bands of left frontal cortex. In addition, both sham and real cTBS increased frontal-parietal phase coupling in the alpha-beta bands concomitant with an increase in amplitude of parietal P50-N70 complex. These data suggest that weak electric fields from sham cTBS can affect both local and downstream neuronal circuits, though in a different manner than high strength TMS.

## Introduction

Non-invasive neuromodulation methods rely on effects produced by a broad range of electric field strengths ranging from 100 mV/mm for transcranial magnetic stimulation (TMS; Miranda et al., 2007; Salvador et al., 2011) to only a fraction of 1 mV/mm for transcranial direct current stimulation or transcranial alternating current stimulation (tDCS/tACS; Datta et al., 2009; Salvador et al., 2010; Miranda et al., 2013). It has recently been shown that low frequency (<8 Hz) extracellular electric fields smaller than 1 mV/mm can cause neuronal entrainment by ephaptic coupling in rat cortical pyramidal neurons (Anastassiou et al., 2011). Additional evidence has demonstrated that electric fields lower than 1 mV/mm can produce effects in hippocampal slices for alternating current (AC) fields having a frequency range from 10 to 100 Hz (Deans et al., 2007) and low-frequency pulsed electric fields (Francis et al., 2003), as well as for direct current (DC) fields <10 and <4 mV/mm (Bikson et al., 2004; Radman et al., 2007). Furthermore, neocortical activity can also be influenced by low-frequency sinusoidal electric fields <0.5 mV/mm (Frohlich and McCormick, 2010; Ozen et al., 2010). Collectively, it is important to realize that weak electric fields from both DC (Nitsche and Paulus, 2000) and AC stimulation (Moliadze et al., 2010) have been shown to transiently alter cortical excitability in humans.

Continuous theta burst stimulation (cTBS) is a repetitive TMS protocol that can induce cortical excitability changes in a short stimulation period (Huang et al., 2005). cTBS typically operates at 70% of individual motor threshold which corresponds to electric field strengths in the range of 50–80 mV/mm. This range of electric field strength is approximately two orders of magnitude higher than what is minimally needed to cause an effect on neurons (Francis et al., 2003). Despite this, the effects of weak electric fields induced by TMS have not been extensively studied. These issues are especially important for TMS studies applying sham protocols. TMS shamming has taken many forms over the years, though currently accepted approaches use coils with magnetic shields that reduce the magnetic field strength and attenuate the induced electric field by about 80–95% depending on the coil type and manufacturer. While it is generally assumed that these sham coils have no neuronal effect (Duecker and Sack, 2013) the induced electric fields are indeed on a range that could affect cortical excitability.

In the present study we investigated how weak electric fields induced by sham TMS over the left dorsolateral prefrontal cortex (DLPFC) exert effects upon local and functionally connected neuronal circuitry using electroencephalographic (EEG) measures of phase connectivity and amplitude of somatosensory evoked potentials (SEPs). To better assess the effect of sham TMS, we included a biologically inert “sham-sham” stimulation condition. In addition, we used computational modeling to evaluate the electric field distribution induced by real and sham TMS. Our observations demonstrate that weak electric fields generated by sham TMS can exert direct neural effects that differ from real TMS as well as the sham-sham condition.

## Materials and methods

### Participants

Fourteen participants (7 male, age 19–61, mean 34.3 ± 16.5 years) were included in the first part of the experiment testing cTBS versus sham stimulation. Fourteen participants (10 male, age 18–38, mean 25.7 ± 6.7 years) were included in the second portion of the experiment testing the effect of a biologically inert (sham-sham) stimulation versus cTBS and sham stimulation. Two participants who took part in the first experiment also took part in the second part of the experiment. All participants provided written informed consent to voluntarily participate in the study and received remuneration for participation. None of the participants reported current drug use (prescription or otherwise) or a history of neurological impairment and all were self-report right hand dominant. All procedures were approved by the Institutional Review Board at Virginia Tech.

### TMS

For the first portion of experiment 1, participants underwent both cTBS and sham TMS protocols on separate days separated by at least 48 h. Both cTBS and sham stimulation was performed with a MagPro X100 Stimulator (MagVenture, Inc., Atlanta, GA, USA) and Cool-B65-A-P-Butterfly-Coil (2 layers of 5 windings at each wing, winding height 12 mm, inner diameter 35 mm, outer diameter 75 mm). The coil was positioned over the international 10–20 electrode site F3 with the coil handle angled 45° outward from midline. A continuous theta burst (cTBS) repetitive protocol was used according to Huang et al. (2005) at 70% of participants' resting motor threshold. Briefly, cTBS consists of 3 single biphasic pulses separated by 0.02 s (50 Hz) repeated every 0.2 s (5 Hz) for a total of 600 pulses delivered in 40 s. The mean resting motor threshold was 55 ± 6% of maximum stimulator output. Sham stimulation was performed with the identical parameters and intensity using the “sham” side of the coil. The sham side of the coil uses a mu metal plate for reduction of the magnetic field from the TMS coil. For both real and sham stimulation, participants wore surface electrodes (Neuroline 710, Ambu Inc. MD, USA) separated by 5 cm, placed on the forehead 3 cm above the left eye. Low current electrical stimulation was delivered through these electrodes in unison with the TMS to help mask and mimic TMS sensation and improve shamming according to recommended procedures. The intensity of the electric pulses was individually set for each participant to a “6 out of 10” on a discomfort scale where 0 was “no feeling,” 5 “slightly uncomfortable” and 10 was “painful cannot continue.” All participants tolerated the real and sham stimulation well.

Individual motor thresholds were determined using a C-B60 coil (figure-8 coil, 2 layers of 5 windings at each wing, winding height 11 mm, inner diameter 35 mm, outer diameter 75 mm). The coil was placed on the left hemisphere over the motor strip with handle position at 45° outward from midline. The coil was moved in 1 cm increments and single pulses were delivered every 5 s and stimulator intensity adjusted until half of the total pulses resulted in a small, but noticeable twitch of the right thumb.

Collection for the second portion (sham-sham) of the experiment was identical to that described above, except that sham-sham stimulation consisted of placing a custom made biologically inert “coil” on the head. This coil was made from wood and metal piping to convey the same physical (pressure) sensation upon the head as both the cTBS and sham stimulation protocols. Participants were unable to view the coil or the stimulation procedure. Participants underwent motor threshold testing as above and were outfitted with the identical forehead electrodes to mimic electrical stimulation as in the first portion of the experiment and were additionally outfitted with earphones that played a recorded audio file of the cTBS protocol to mimic auditory stimulation from both cTBS and sham stimulation procedures.

### EEG

Electroencephalographic (EEG) data were acquired using a DC amplifier (BrainAmp MR Plus, Brain Products GmbH Gilching Germany) with an acticap 64 channel cap (Brain Products GmbH), referenced to the average of 64 channels.

Data were sampled at 1000 Hz and online filtered at DC-250 Hz. Impedances of all electrodes were confirmed to be <5kΩ. Somatosensory evoked potentials (SEPs) were derived from electrical stimulation of the median nerve of the right wrist using square wave pulses of 0.5 ms duration (GRASS SD9 stimulator, West Warwick, Rhode Island, USA) delivered through a surface bar electrode (2 cm electrode spacing), anode distal, fixed to the wrist using tape. The intensity of the pulse was adjusted until a slight but noticeable thumb twitch was achieved. Electrical pulses were delivered at an interstimulus interval of 1.2 s with a positive randomization of 1.5 s. A total of 250 stimuli were delivered for each session. Stimulation intensity was monitored via surface electromyography (EMG) of the thumb using a SX230 EMG sensor (1 cm diameter, 2 cm spacing) and K800 amplifier (Biometrics Ltd, Ladysmith Virginia USA) and sampled at 100 Hz using Spike2 version 7.08a software (CED, UK) and stored on a computer for later analysis. EEG data were collected prior to the cTBS/Sham intervention (Pre) and at two time points post.

cTBS/Sham stimulation (Post1: 5–7 min; and Post2: 25 min). During median nerve stimulation participants were required to fixate on a cross on a computer screen placed in front of them. Total testing time for each EEG recording session was roughly 6 min.

#### EEG—weighted phase lag index

EEG data were processed using custom Matlab scripts (Matlab - The Mathworks, Inc., Natick, MA), EEGLAB (Delorme and Makeig, 2004) and Fieldtrip (Vinck et al., 2011). For all EEG analyses we focused on the pre stimulation and 25 min post stimulation data to evaluate the effect of the sham stimulation. This was chosen as there was a gradual buildup over time (see Supplementary Figure 1 for an exemplary illustration) with maximal effect in Post2. In the following analysis we refer to the Post2 recording as Post condition. Data were re-referenced to the average of 64 channels, band-pass filtered (1–90 Hz) and notch filtered at 60 Hz. Data were inspected for artifacts using automatic rejection criteria of absolute peak-to-peak amplitude of 100 μV and 80 μV/ms using channels Fp1, Fp2, Af3, Af4, F3, and Cp3. Data were epoched around median nerve stimulus onset (−200 to 500 ms) and baseline corrected (−200 to 0 ms). To assess the effect of both cTBS, sham and sham-sham TMS on local and downstream connected neuronal regions, phase synchronization between local prefrontal areas as well as long range prefrontal and somatosensory brain areas was computed using the weighted phase lag index (WPLI) (Vinck et al., 2011) which is unaffected by volume conduction. WPLI was computed between local channel pairs F3-F1, F3-F5, F3-FC1, F3-FC3, and F3-FC5 as well as F3-CP3, F3-CP4, F3-F4, and CP3-CP4. Differences in phase synchronization were computed between Post and Pre stimulation for all three conditions (cTBS, sham and sham-sham). Statistical analysis of the Post-Pre WPLI was performed using non-parametric cluster threshold analysis (Maris and Oostenveld, 2007) with 10,000 repetitions, initial cluster threshold *p* = 0.05 for the time points between -100 and 400 ms and frequency bands 7–13 and 14–30 Hz and FDR correction (Benjamini–Hochberg, false discovery rate *q* = 0.05) for number of channel combinations and conditions. This procedure was conducted separately for channel combinations starting from channel CP3 (3 combinations × 3 conditions) and channel combinations starting from channel F3 (7 combinations × 3 conditions). For cTBS vs sham a dependent *t*-test was conducted, and for cTBS vs. sham-sham, as well as sham vs. sham-sham, an independent *t*-test was conducted. To control for potential existing between group differences in EEG, we also tested for possible baseline differences between groups for the three Pre conditions using the same procedure as for the Post-Pre comparisons.

#### EEG—somatosensory evoked potentials

Data were preprocessed as above and epoched around median nerve stimulation onset (-100 to 300 ms) and baseline corrected (−100 to 0 ms). Peak to peak amplitudes were quantified from channel F3 and CP3. For channel F3 potentials included P14-N30, N30-P45, P45-N60, N60-P80, and P80-N100. For channel CP3, potentials included P14-N20, N20-P27, N27-N33, N33-P50, P50-N70, N70-P100, and P100-N140. Potentials were quantified for each participant for each stimulation procedure (cTBS, sham, sham-sham) for both the Pre and Post conditions. A One-Way independent samples analysis of variance (ANOVA) was conducted for each of the potentials from channel F3 and CP3 on the Post-Pre data with factors (cTBS, sham, sham-sham). Significant (*p* < 0.05) *F*-tests were evaluated with *post-hoc* Tukey–Kramer tests.

### Magnetic field mapping

The magnetic field of the real and sham side of the TMS coil was measured using a custom built magnetic probe made of two rectangular shaped windings of wires (1 cm^2^ surface area) that were oriented perpendicularly to each other. A grid of 19 × 19 points (1 cm spacing) was placed directly on the coil face to measure the magnetic field induced voltages in x and y direction for both the real and sham face of the TMS coil. A computational model of the TMS coil that reproduces the spatial pattern of the measured magnetic field was constructed using a dipole approximation (Thielscher and Kammer, 2004). Small magnetic dipoles are positioned at the coil windings and the total magnetic vector potential is obtained by summing up individual magnetic dipoles. For the sham coil side, the effect of the magnetic shield was mimicked by increasing the distance of the coil to the measurement plane in the computational model such that the computational and measurement data match each other.

### Electric field simulations

Electric field simulations were performed for both the active and sham side of the TMS coil. A finite element model of the head for one subject was constructed using Simnibs (Windhoff et al., 2013). From T1- and T2- weighted MR images five different tissue types WM, GM, CSF, skin and skull were segmented and a FEM model consisting of ca. 1.7 million tetrahedral elements was constructed. TMS coil position and orientation over the left DLPFC was recorded using a neuronavigation system (Visor 2, ANT). For more detailed methods about the FEM simulations see (Opitz et al., 2011, 2013; Windhoff et al., 2013).

## Results

### Magnetic field measurements and electric field simulations

**Figure 1:**
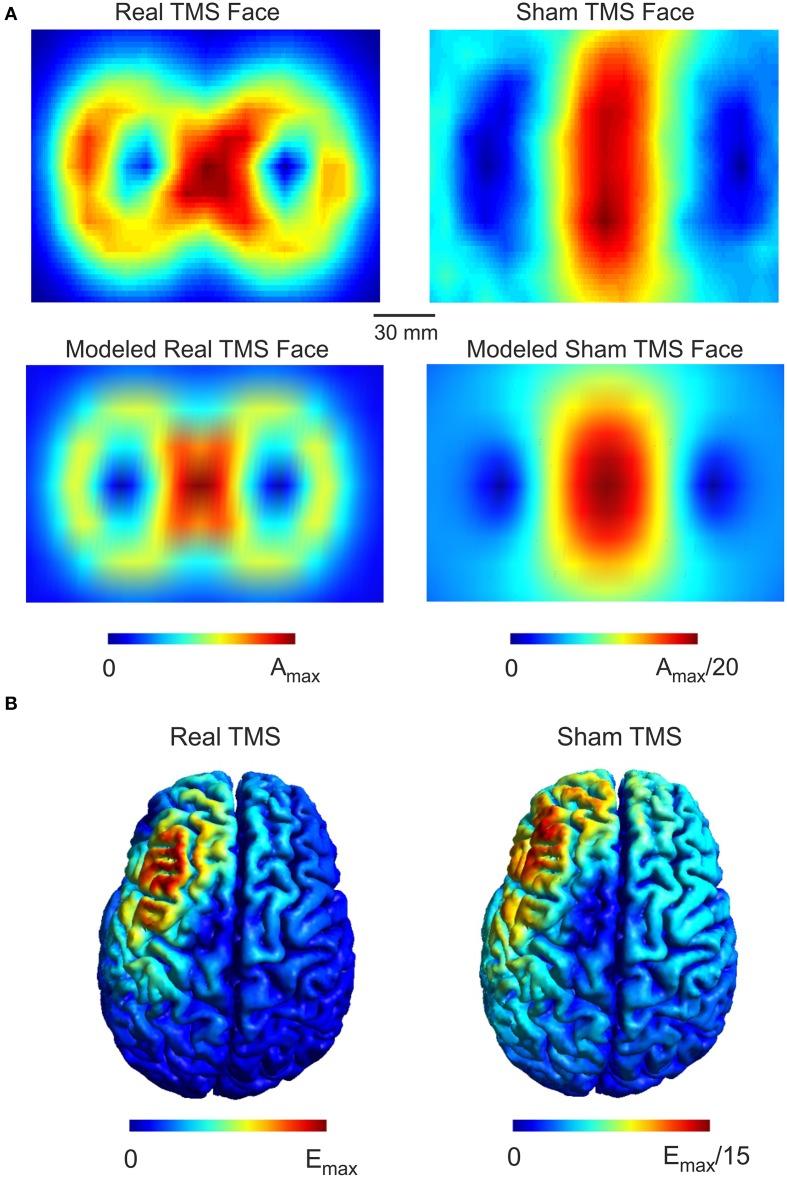
**Real and Sham TMS fields. (A)** Distributions of the magnetic vector potential for the real and sham side of the TMS coil. The upper panels illustrate measured values for both real and sham faces of the coil while the lower panels illustrate the modeled field distribution. The magnetic vector potential for the real side is about 20 times stronger than for the sham side. The coil models faithfully capture the measured field distribution as illustrated. **(B)** The simulated electric field distributions generated by real and sham cTBS of the left DLPFC. The electric field strength generated by real TMS is about 15 times stronger than the electric field strength produced by sham TMS. The spatial distributions of the electric field generated by real and sham TMS were strikingly similar to one another.

The sham side of the TMS coil produced a magnetic field maximum approximately 20-fold less than the real side (Figure 1A). The field distribution of the real side has a shape typical of figure-8 coils with highest field strength at the coil center and moderate field strengths at the wings of the coil. The sham side of the coil exhibited a broad area of high field strength only in the center region of the coil. The computational model of the TMS coil was able to accurately capture the general distribution of the measured magnetic field (Figure 1A). The root mean square error between the measured and modeled magnetic field was 7.7 and 7.5% in the central region of the coil (1 × 1 cm) for the real and sham sides respectively. In one circumscribed region at the coil wing the error was up to 50% which was likely due to a small asymmetry in the coil with regard to the measurement plane. However, as the electric field distribution is mainly driven by the center of the coil this was unlikely to significantly influence the accuracy of the electric field simulations.

While the maximum magnetic field of the sham coil side was reduced 20-fold, the maximum induced electric field was only reduced 15-fold (Figure 1B). Furthermore, the electric field distribution was broader for the sham condition. The broader distribution of the sham side is due to the smearing effect of the magnetic shield, which defocusses as well as reduces the magnetic and the electric field.

### Stimulation effects on phase synchrony

**Figure 2:**
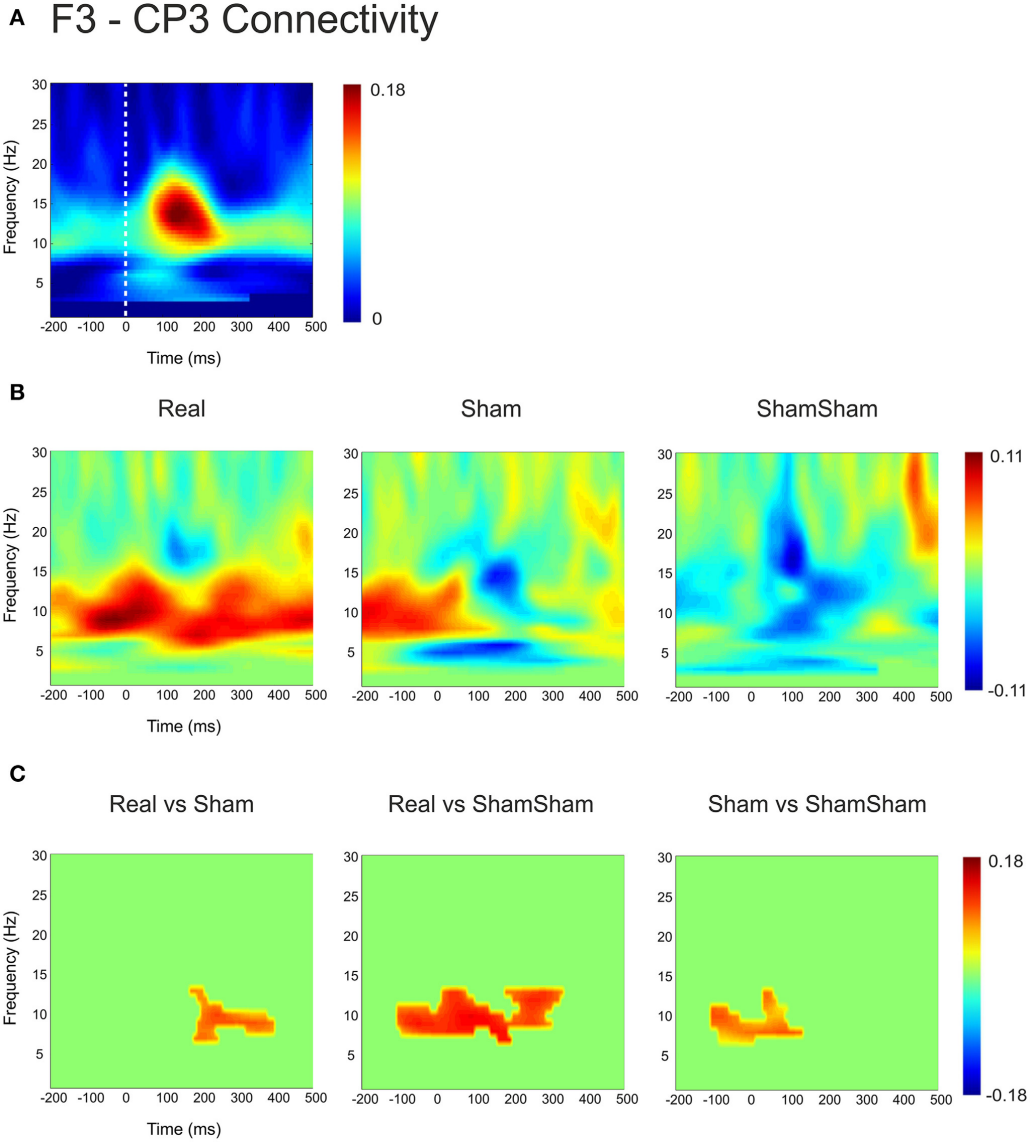
**Quantification of changes in cortical EEG connectivity. (A)** The baseline weighted phase lag index (WPLI) for the F3-CP3 channel pair during median nerve stimulation is shown. The *dashed lines* indicate the onset of the median nerve stimulus. An increase in connectivity in the high alpha, low beta range is visible after the stimulus onset. **(B)** Connectivity changes (Post-Pre) are shown for real, sham and sham-sham stimulation. Differential effects on connectivity changes are visible depending on stimulation condition. **(C)** Significant differences between the connectivity changes in real vs. sham, real vs. sham-sham, as well as sham vs. sham-sham are shown. Strongest effects were present for real vs. sham-sham, with a strong increase in the WPLI in the alpha and low beta band in the real condition.

**Figure 3:**
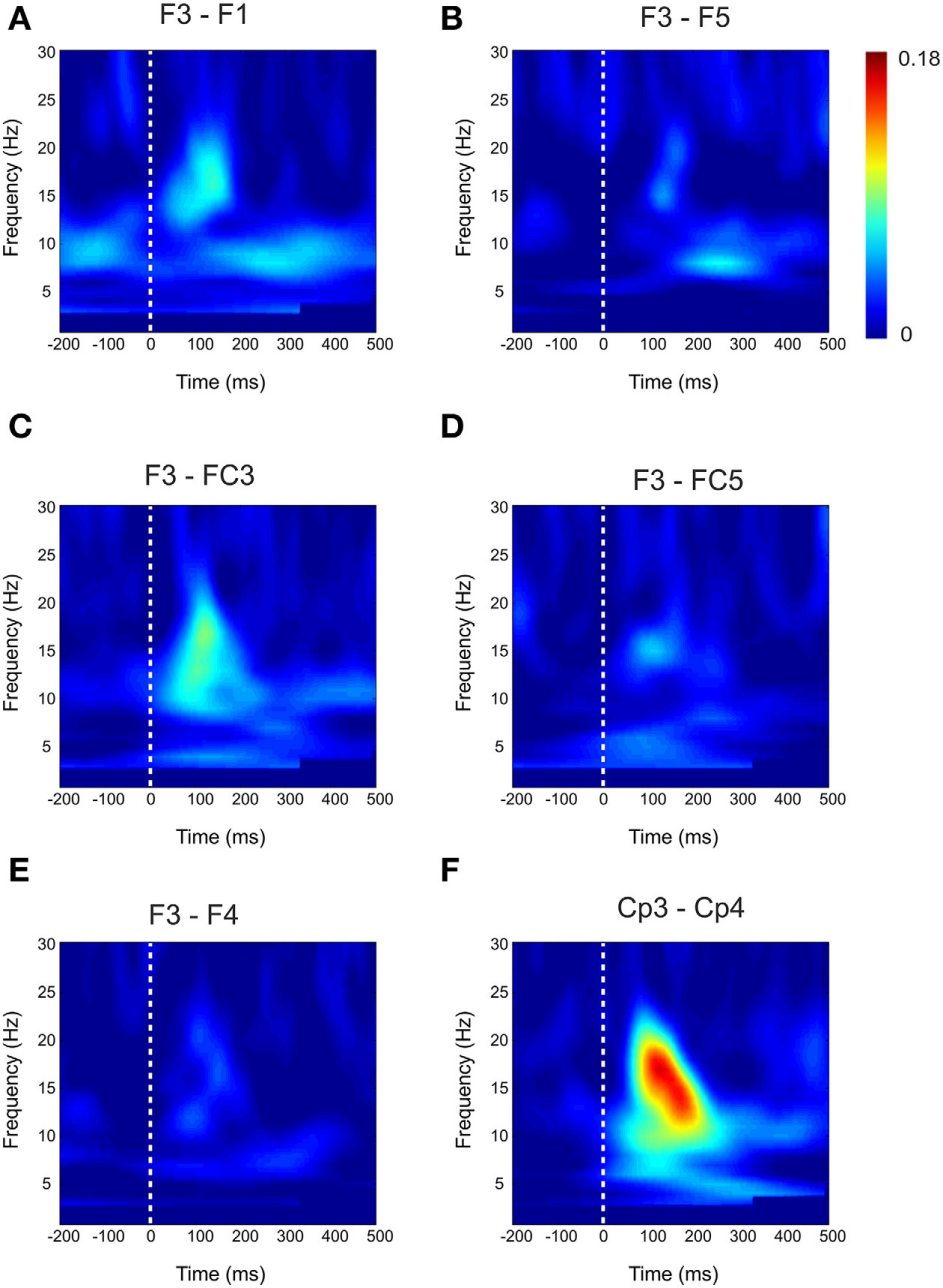
**Baseline Connectivity for local and interhemispheric connections**. Baseline weighted phase lag index (WPLI) during median nerve stimulation for local channel connections **(A–D)** and interhemispheric connections **(E–F)**.

Based on the observations described above, we next examined the functional connectivity between local frontal electrodes as well as downstream connections using the weighted phase lag index (WPLI) as a measure of phase synchronization. Of special interest was the effect between the stimulation site F3 and the localization site of parietal somatosensory evoked potentials recorded from electrode site CP3 based upon previous research demonstrating this connection (Yamaguchi and Knight, 1990). Under baseline, the WPLI showed a significant increase in alpha and beta (10–20 Hz) coupling between CP3 and F3 immediately after median nerve stimulation (*p* < 0.025) (Figure 2A). This CP3-F3 phase synchronization was considerably stronger than local F3 channel pairs (*p* < 0.025) (Figure 3). After real cTBS the WPLI showed a broad increase for alpha and beta frequencies (*p* < 0.025) (Figure 2B). After sham cTBS we observed a decrease in beta as well as theta activity occurring at 100–200 ms (Figure 2B). For sham-sham a broad decrease in the WPLI after median nerve onset could be seen in the Post-condition compared to baseline (Figure 2B). Individual comparisons showed significant effects between all conditions (Figure 2C). A strong enhancement of phase coupling in the alpha-beta frequency range (7–14 Hz) was found comparing cTBS with sham-sham (*p* < 0.0083) (Figure 3C middle panel). Similar effects, yet for shorter time windows, were found for the cTBS vs. sham and sham vs. sham-sham comparisons (Figure 2C). Most importantly, sham cTBS showed significant increases in phase coupling in the alpha-beta frequency compared to the sham-sham condition (*p* < 0.0083). These data indicate that both cTBS and sham cTBS can induce lasting changes amongst cortical networks which differ from a biologically inert control condition. There were no significant differences in the baseline connectivity between stimulation conditions (*p* > 0.05).

**Figure 4:**
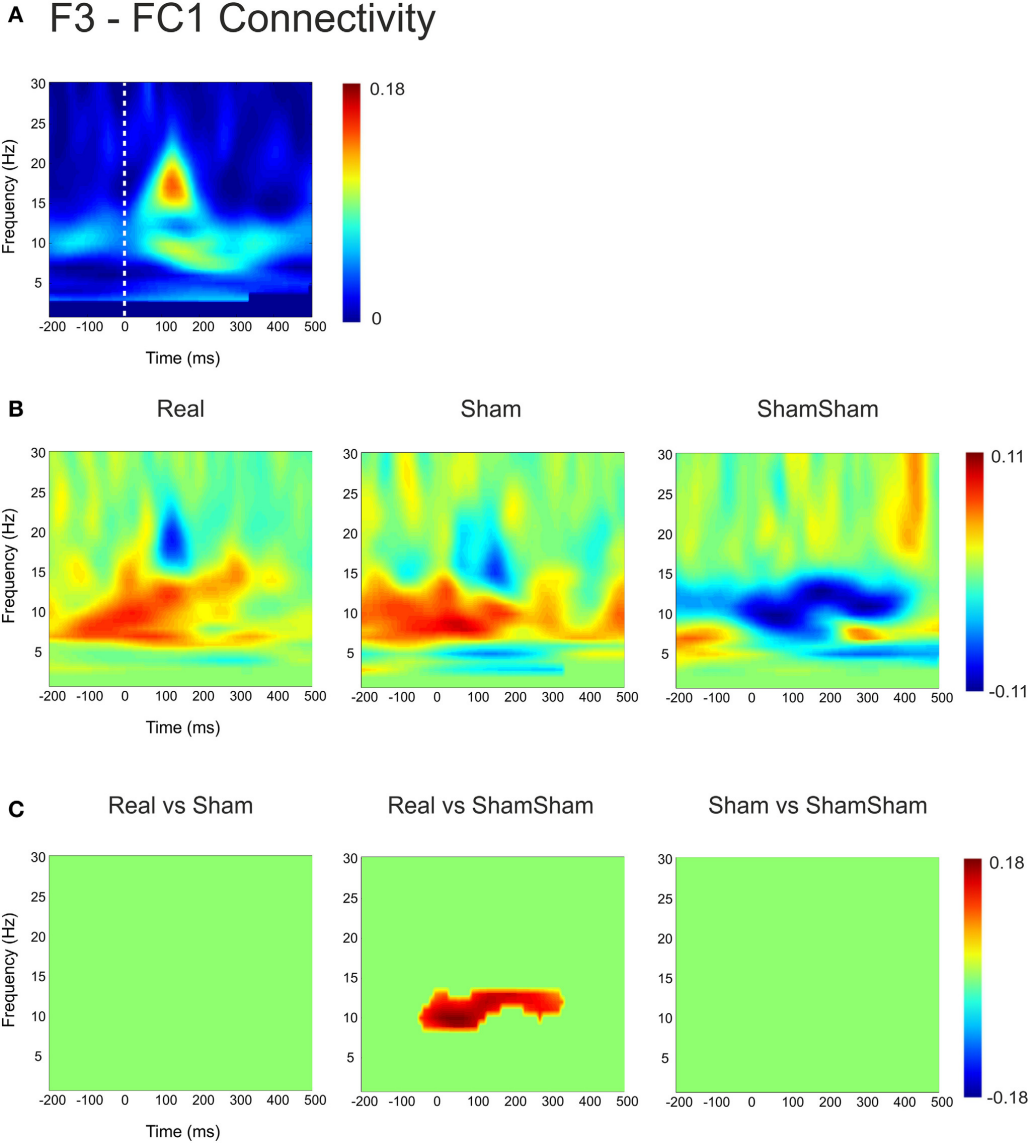
**Connectivity changes in F3-FC1. (A)** Baseline weighted phase lag index (WPLI) during median nerve stimulation. **(B)** Connectivity changes (Post-Pre) for real, sham and sham-sham stimulation. **(C)** Significant differences between the connectivity changes in real vs. sham, real vs. sham-sham and sham vs. sham-sham.

**Figure 5:**
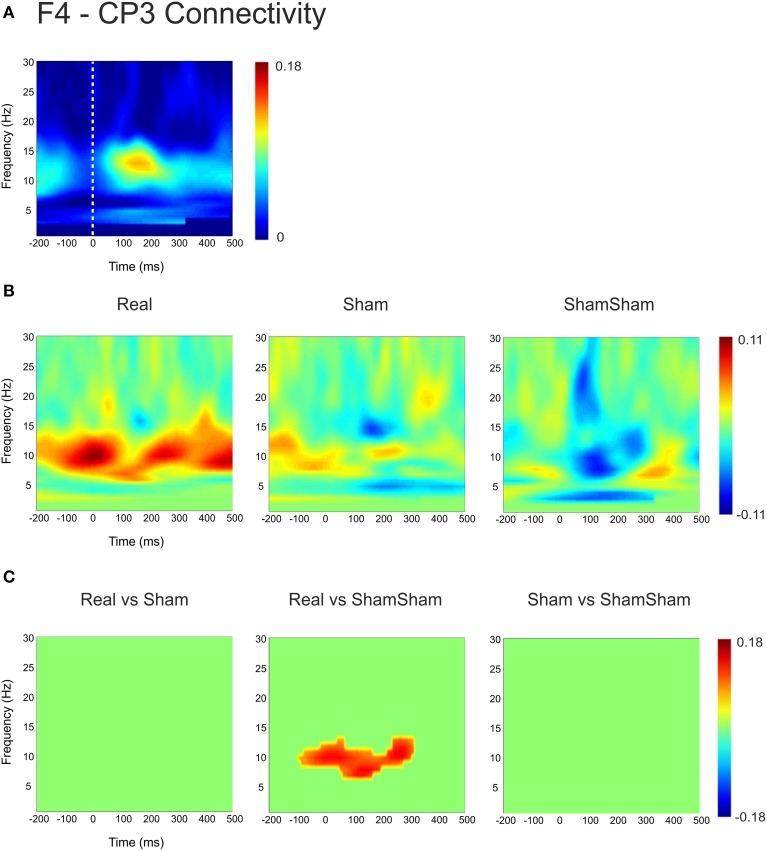
**Connectivity changes in F4-CP3. (A)** Baseline weighted phase lag index (WPLI) during median nerve stimulation. **(B)** Connectivity changes (Post-Pre) for real, sham and sham-sham stimulation. **(C)** Significant differences between the connectivity changes in real vs. sham, real vs. sham-sham and sham vs. sham-sham.

**Figure 6:**
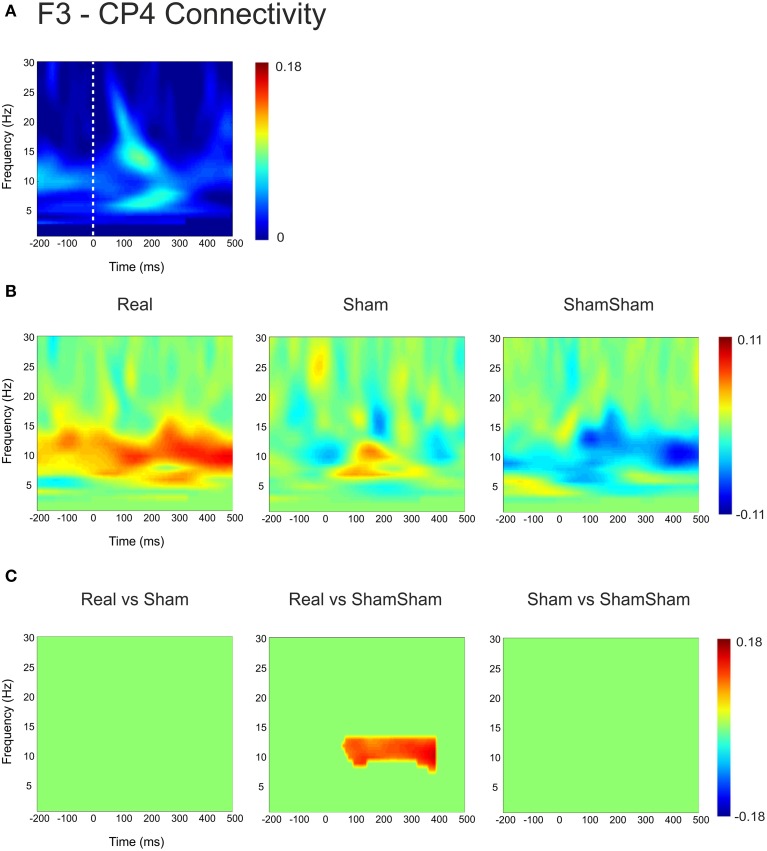
**Connectivity changes in F3-CP4. (A)** Baseline weighted phase lag index (WPLI) during median nerve stimulation. **(B)** Connectivity changes (Post-Pre) for real, sham and sham-sham stimulation. **(C)** Significant differences between the connectivity changes in real vs. sham, real vs. sham-sham and sham vs. sham-sham.

For the local channel pair F3-FC1, a similar effect as for F3-CP3 was found for real vs sham-sham (*p* < 0.0083) (Figure 4), however no effects were found for the other comparisons. We did not observe any stimulation related changes in cross-hemispheric phase synchronization between F3-F4 and CP3-CP4 despite strong intrinsic coupling between CP3 and CP4 in the alpha-beta frequency range (Figure 3). Interestingly, stimulation effects were apparent between F4-CP3 and F3-CP4 (Figures 5, 6) with a significant difference (*p* < 0.0083) between real and sham-sham.

### Somatosensory evoked potentials

**Figure 7:**
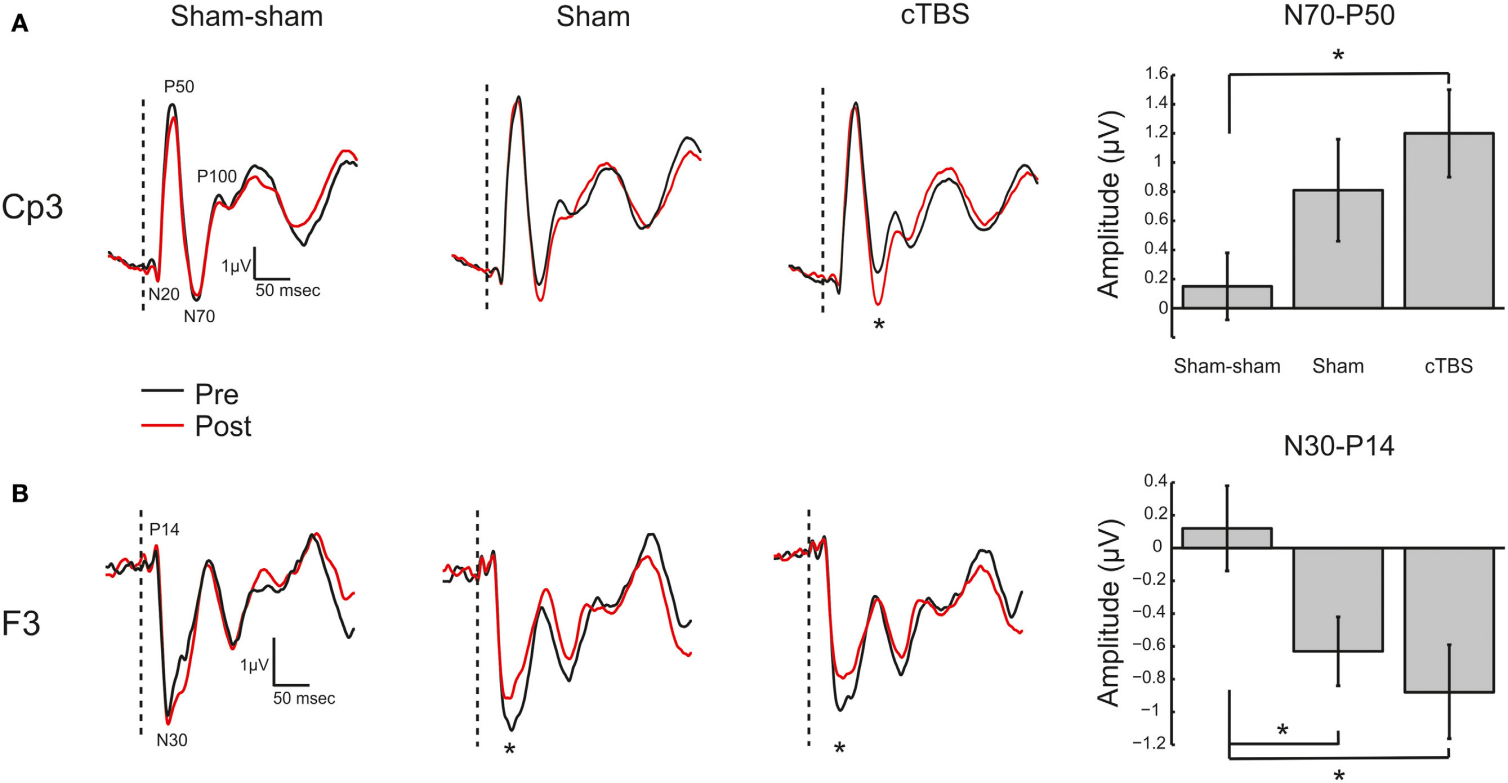
**Somatosensory Evoked Potentials. (A)** Left: Grand average (*N* = 14) somatosensory evoked potential (SEP) traces recorded from electrode site CP3 for the three TMS conditions (Sham-sham, Sham, cTBS). Potentials of interest are marked on trace. The vertical dashed bar represents onset of median nerve stimulation. Black trace (Pre) is before TMS intervention and red trace (Post) is after TMS intervention. ^*^Denotes significant amplitude difference (*p* < 0.05). Right: Grand average (*N* = 14) N70 to P50 peak to peak amplitude in microvolts (μV) with ± SEM. ^*^Denotes *p* < 0.05. **(B)** Left: Grand average (*N* = 14) SEP traces recorded from electrode site F3. Potentials of interest are marked on trace. ^*^Denotes significant amplitude difference (*p* < 0.05). Right: Grand average (*N* = 14) N30 to P14 peak to peak amplitude in microvolts (μV) with ± SEM. ^*^Denotes *p* < 0.05.

Somatosensory evoked potential peak to peak amplitudes were quantified from channel F3 and CP3. For channel CP3 a significant effect was found for the N70-P50 SEP complex [*F*_(2, 39)_ = 3.27, *p* = 0.0491]. A *post-hoc* Tukey–Kramer test revealed a significant increase (*p* < 0.05) in amplitude of the N70-P50 for cTBS compared to sham-sham only (Figure 7A). No statistically significant differences were found for any of the other quantified potentials (Table 1). For channel F3 a significant decrease in amplitude was found for the P14-N30 potential [*F*_(2, 39)_ = 4.04, *p* = 0.0258]. *Post-hoc* Tukey–Kramer tests revealed a significant decrease in amplitude of the peak to peak P14-N30 potential for cTBS compared to sham-sham (*p* < 0.05), and for a significant decrease in amplitude for sham compared to sham-sham (*p* < 0.05) (Figure 7B and Table 1). No statistically significant differences were found for any of the other potentials quantified from channel F3 (Table 1).

**Table 1:** **Somatosensory evoked potential amplitudes**.

	**cTBS**	**Sham**	**SS**	***P*-value**
**CP3**				
N20-P14	−0.19 (0.09)	−0.10 (0.07)	0.06 (0.07)	0.22
P27-N20	−0.18 (0.08)	−0.03 (0.11)	−0.18 (0.21)	0.84
N33-P27	−0.06 (0.15)	−0.01 (0.10)	0.02 (0.12)	0.95
P50-N33	−0.18 (0.15)	0.18 (0.49)	−0.10 (0.21)	0.79
N70-P50	1.20 (0.30)	0.81 (0.35)	0.15 (0.23)	**0.0491**
P100-N70	−0.16 (0.48)	0.08 (0.26)	0.03 (0.36)	0.89
N140-P100	0.28 (0.27)	0.26 (0.37)	0.30 (0.34)	0.99
**F3**				
N30-P14	−0.88 (0.29)	−0.63 (0.21)	0.12 (0.26)	**0.0258**
P45-N30	−0.36 (0.42)	0.16 (0.25)	0.02 (0.19)	0.47
N60-P45	−0.32 (0.33)	0.19 (0.24)	0.02 (0.13)	0.36
P80-N60	−0.62 (0.52)	−0.46 (0.33)	−0.21 (0.29)	0.77
N100-P80	−1.13 (0.46)	−0.91 (0.56)	−0.33 (0.26)	0.46

*Group average (mean and SEM, N = 14) peak-to-peak amplitudes in μV from channel CP3 (top) and F3 (bottom). cTBS, real stimulation; Sham, weak field stimulation; SS, sham-sham*.

*Bold values highlight significant effects (p-value < 0.05) in the Anova*.

## Discussion

We investigated the effects of weak electric fields induced by sham cTBS to the left DLPFC on EEG phase connectivity and SEP amplitudes. Results demonstrate that both real and sham cTBS over the left DLPFC increase both local and down-stream phase-coupling in the alpha-beta (7–14 Hz) frequency range and both decrease amplitude of frontal SEPs but only real cTBS to significantly increase amplitude of parietal SEPs. The results of this study provide evidence that low electric field strengths from short duration cTBS can have an influence on local and downstream cortical activity.

tACS and tDCS produce electric fields in the brain roughly an order of magnitude less than sham TMS as used here. It should not be too surprising then to find effects upon somatosensory activity from sham stimulation given that tACS (Feurra et al., 2011) and tDCS (Matsunaga et al., 2004; Dieckhofer et al., 2006) also affects somatosensory processing. Here, weak stimulation was delivered over the frontal cortex, yet changes were recorded in SEP potentials generated in somatosensory areas in the parietal cortex (Allison et al., 1991). This is likely due to anatomical (Pandya and Barnes, 1987) and functional (Yamaguchi and Knight, 1990) connectivity between the dorsolateral pre-frontal cortex and somatosensory areas. Yamaguchi and Knight (1990) showed that lesions of the DLPFC resulted in altered amplitude of early SEPs recorded from primary somatosensory cortex (S1) (Yamaguchi and Knight, 1990). The strong baseline phase connectivity between electrodes sites F3 and CP3 confirm this. As such, we hypothesized that transient inhibition of the left DLPFC from high field cTBS should alter SEP amplitudes measured from S1 similar to the lesion results. This was indeed the case, and sham cTBS, which induced electric field amplitudes about 5% of that produced by real TBS, trended toward the same effect. It is possible that low electric fields have weaker effects on downstream connected regions and may exert their effect mainly on local circuits within the maxima of the induced electric field. The SEP data from electrode site F3 support this. There was a significant difference in potential amplitude between sham stimulation and sham-sham stimulation only at site F3 which is supported by the phase connectivity results. Thus, there seems to be a graded response with respect to stimulation strength with strongest effects for real cTBS and weaker but not negligible effects for sham cTBS. Interestingly, the direct comparison of real versus sham stimulation did not result in significant phase synchrony effects for many channel pairs. This was not due to a missing stimulation effect or non-specific effects due to habituation to the task over time. On the contrary, both real and sham cTBS showed stimulation induced changes in a similar direction compared to the sham-sham condition. The effect of weak electric fields delivered to DLPFC upon somatosensory processing is not unprecedented. Bolton and Staines (2011) also reported that their sham stimulation resulted in an effect upon parietal SEPs. We estimate the electric field induced by their sham condition (6% resting motor threshold) (Bolton and Staines, 2011) induced an electric field in the left frontal cortex at around the same order of magnitude as that applied in this study. Thus, low electric field strength produced by sham stimulation exerts differential effects upon excitability in cortical areas directly under the TMS coil where the electric field is largest but also crucially can affect downstream anatomically and functionally connected regions. While not being the main focus of our study the effects of cTBS increased over time and we subsequently selected the post stimulation timepoint after 25 min to evaluate the effects of real and sham stimulation. Stronger stimulation effects are not uncommon in brain stimulation protocols. For example in one study using tDCS to induce plasticity in the motor cortex strongest effects on motor evoked potentials have been found after 30 min (Kuo et al., 2013). Also in the original paper introducing theta burst stimulation (TBS) (Huang et al., 2005) there were larger effects or effects at least as strong after 20 min compared to the immediate after effect of the stimulation.

Studies of slight TMS intensity differences also report correspondingly varying effects: cTBS of the motor cortex at 65% of resting motor threshold decreased motor excitability while 70% increased it (Doeltgen and Ridding, 2011). Similar intensity dependent effects have been found for tACS using 140 Hz over M1 (Moliadze et al., 2012), such that 0.4 mA reduced the amplitude of MEPs while 1.0 mA enhanced them. Also, a reversal of stimulation effects from cathodal tDCS stimulation was found from inhibition to excitation by changing intensities from 1 to 2 mA (Batsikadze et al., 2013). A possible mechanism for a reversal in the direction of effects using different intensities might be that inhibitory neurons are more rapidly excitable than excitatory neurons by lower field strengths. For higher field strengths excitatory neurons are more strongly activated and prevail over inhibitory effects that lead to a net excitatory effect (Berger et al., 2011; Herrmann et al., 2013). We found differential effects on SEP amplitudes both at the site of stimulation and in S1 concomitant with differences in connectivity between F3 and CP3 for sham and real cTBS.

**Figure 8:**
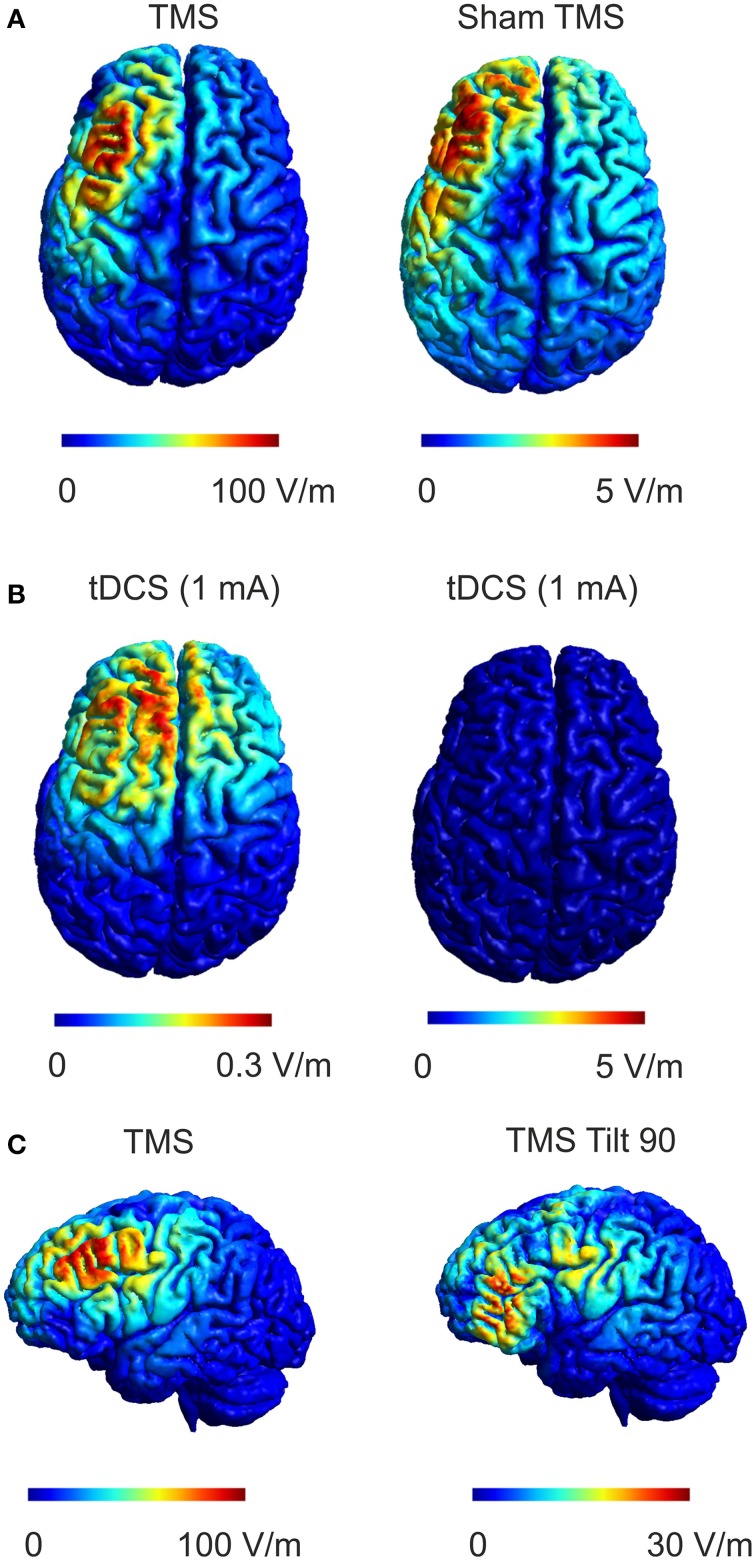
**Comparison of real and sham TMS induced electric fields with electric fields during tDCS**. **(A)** The electric field distribution in the brain for real TMS (left panel, dI/dt = 70 A/μs which corresponds to the mean rate of change of coil current employed in the study, *left*) and sham TMS (right panel) is shown. **(B)** Electric field produced by 1 mA tDCS using a 4 × 4 cm anode and a 5 × 7 cm cathode positioned over the DLPFC and contralateral supraorbital orbit, respectively are shown for two different scales. Comparing sham TMS to tDCS, the sham TMS electric field is about 15 times stronger than the tDCS electric field while exhibiting a similar spatial distribution. **(C)** The electric field distribution in the brain for real TMS (as in **A**, left panel) compared to the electric field distribution for another commonly used sham method implemented by tilting the coil 90° away from the head (right panel). The induced electric field strength by tilting the coil 90° can reach up to 30% of the strength during normal TMS.

Sham approaches for TMS have been investigated and constantly improved over the last decade. Early sham approaches were implemented by tilting the TMS coil away from the head, which was shown to induce significant residual electric fields (Loo et al., 2000; Lisanby et al., 2001). For example tilting the TMS coil 90° away from the head can still induce electric field strengths that are up to 30% compared to the standard coil position (Figure 8C). Current sham approaches are much more sophisticated, using a combined real or sham coil (Rossi et al., 2007) that can be electronically switched (Deng and Peterchev, 2011), concomitant audio stimulation and surface electrodes attached to the head that deliver electric current in unison with TMS pulsing to help ensure that skin sensations are identical between conditions. These approaches are very effective at disguising the appearance and accompanying sensory stimulation associated with TMS and are important features needed to investigate effects between real and sham stimulation only due to the direct effect of the induced electric field. However, in our opinion, not enough attention has been given to the residual electric field that is induced in the brain during sham treatments. While magnetic shields reduce the electric field effectively to about 5% of its strength during real stimulation, these fields are still in the range that has been demonstrated to induce lasting effects on neuronal populations. The sham TMS electric field is about an order of magnitude stronger than that produced by 1 mA tDCS (Figures 8A,B) that has proven to produce both cortical excitability and behavior changes in several studies (Fecteau et al., 2007; Holland et al., 2011; Ruff et al., 2013). Although the temporal course of the electric fields is usually different between electrical and magnetic stimulation methods and different protocols cannot easily be directly related to each other, this comparison shows that electric fields occurring during sham TMS are clearly within the range where physiological effects are possible. While the study of effects of weak electric fields induced by TMS is an interesting topic in itself, it is not desirable to have direct neuronal effects of sham stimulation during cognitive studies or especially clinical trials, since the sham condition could indeed be having an effect upon neuronal excitability. Thus the comparison of real and sham effects is made explicitly difficult especially in the case of missing effects between real and sham stimulation. Such observations taken at face value may lead one to conclude that a particular sham controlled TMS intervention was not effective when indeed they both were. It should be noted that this study employed two different subject groups such that possible between groups effects might interfere with our results. However, the baseline connectivity did not significantly differ between groups, as well as the analysis of pre-post changes that control for non-specific effects make this unlikely. These non-specific effects (for example anticipation of getting stimulated) may also contribute to effects between conditions though we were sensitive to keeping sensory stimuli (coil pressure on the head, auditory stimulation) the same during sham and sham-sham stimulation. Furthermore, all subjects in all conditions were naïve to TMS and did not know what to expect if anything across repeated sessions thus limiting the possibility that results were due to non-specific effects. Finally, these data are the result of a single commercial sham-coil using a cTBS rTMS protocol and may not directly transfer to other commercial sham-coils due to differences in the technology used to achieve shamming or to other rTMS protocols. Future studies should address these effects across other sham coils and TMS protocols.

## Conclusions

Sham procedures are absolutely necessary to control for expectation effects which can manifest themselves on a physiological level (Büchel et al., 2014; Fiorio et al., 2014). It is clear that sham TMS, as employed here, produces electric fields in the human cortex of a magnitude that can affect cortical excitability. It is conceivable that the effect of real TMS stimulation may be underestimated in studies comparing it to current shamming protocols. A sham condition that mimics expectation and sensation but does not induce *any* electric field in the cortex is optimal for assessing effects of TMS, but, until this becomes common practice, it is our opinion that the weak electric field induced by current sham TMS approaches should be accounted for or at least considered when interpreting results. Nevertheless, we cannot readily generalize our findings for other rTMS protocols and future research has to investigate whether effects of weak TMS might occur for them as well.

### Conflict of interest statement

The authors declare that the research was conducted in the absence of any commercial or financial relationships that could be construed as a potential conflict of interest.

## Supplementary material

Supplementary Figure 1 **Buildup effect for changes in cortical EEG connectivity after cTBS. (A)** The weighted phase lag index (WPLI) for the F3-CP3 channel pair during median nerve stimulation is shown for three different timepoints (Pre, Post1, and Post2). The WPLI shows incremental increases in the high alpha, low beta range over the two post stimulation timepoints. **(B)** Connectivity changes Post1-Pre (left panel) and Post2-Pre (right panel) are shown to illustrate the effect of increased differences over time.
