# Supplementary figures and images for: Is sham cTBS real cTBS? The effect on EEG dynamics
Source: Front Hum Neurosci. 2015 Jan 8;8:1043. doi: 10.3389/fnhum.2014.01043 (PMC4287020; doi:10.3389/fnhum.2014.01043)

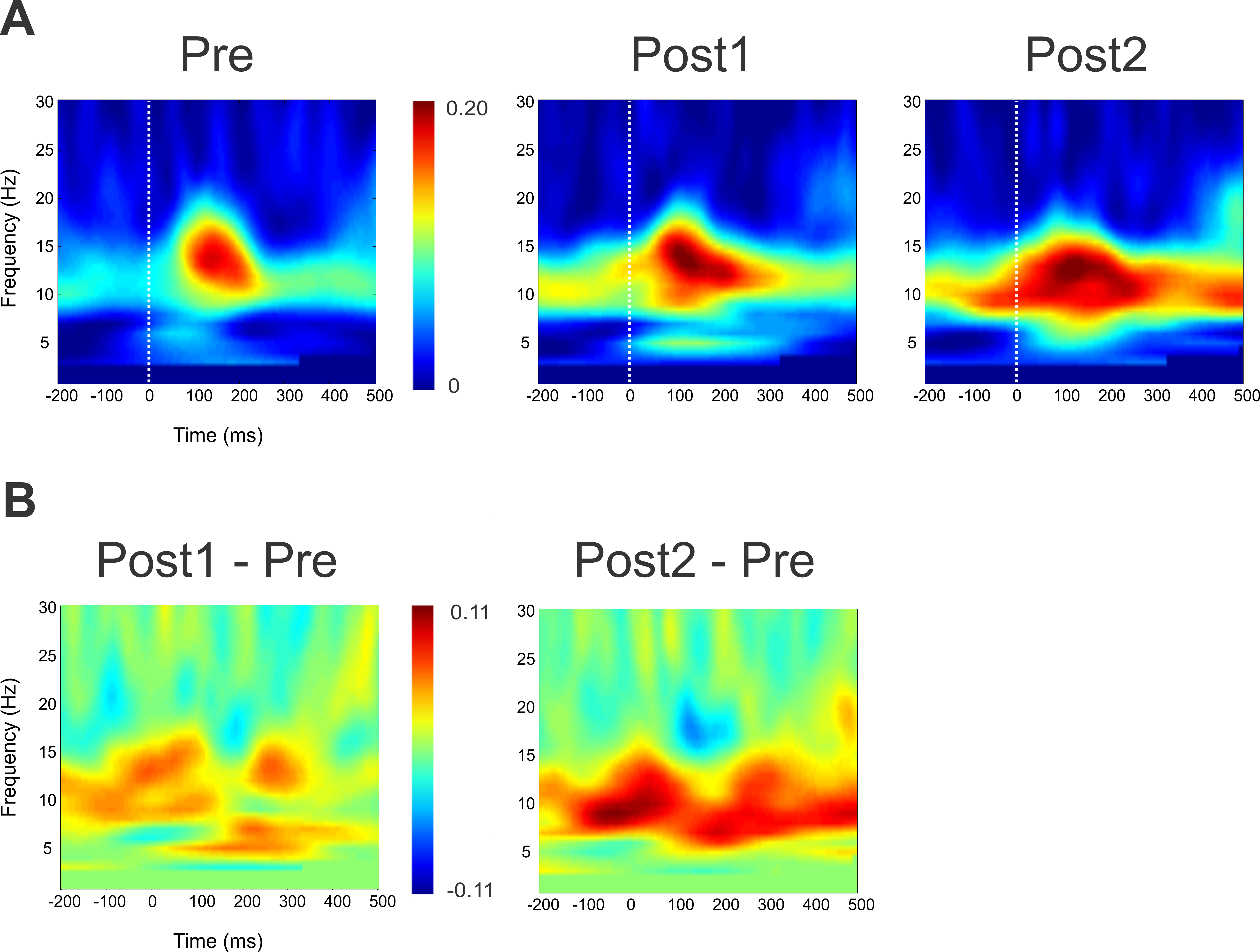

Supplement: Supplementary Figure 1 — Buildup effect for changes in cortical EEG connectivity after cTBS. (A) The weighted phase lag index (WPLI) for the F3-CP3 channel pair during median nerve stimulation is shown for three different timepoints (Pre, Post1, and Post2). The WPLI shows incremental increases in the high alpha, low beta range over the two post stimulation timepoints. (B) Connectivity changes Post1-Pre (left panel) and Post2-Pre (right panel) are shown to illustrate the effect of increased differences over time. [file Image1.TIF]
